# Potential Impact of Antiviral Drug Use during Influenza Pandemic

**DOI:** 10.3201/eid1109.041344

**Published:** 2005-09

**Authors:** Raymond Gani, Helen Hughes, Douglas Fleming, Thomas Griffin, Jolyon Medlock, Steve Leach

**Affiliations:** * Health Protection Agency, Salisbury, Wiltshire, United Kingdom;; † Royal College of General Practitioners, Harborne, Birmingham, United Kingdom

**Keywords:** Epidemic Modeling, Antiviral treatment, Pandemic Influenza, Public Health Planning, Real Time Modeling, research

## Abstract

The recent spread of highly pathogenic strains of avian influenza has highlighted the threat posed by pandemic influenza. In the early phases of a pandemic, the only treatment available would be neuraminidase inhibitors, which many countries are considering stockpiling for pandemic use. We estimate the effect on hospitalization rates of using different antiviral stockpile sizes to treat infection. We estimate that stockpiles that cover 20%–25% of the population would be sufficient to treat most of the clinical cases and could lead to 50% to 77% reductions in hospitalizations. Substantial reductions in hospitalization could be achieved with smaller antiviral stockpiles if drugs are reserved for persons at high risk.

## Abstract 2

Impact of different antiviral treatment strategies on hospitalizations during an influenza pandemic is evaluated.

Recent outbreaks of highly pathogenic avian influenza in poultry in East Asia (H5N1), Canada (H7N3), and the Netherlands (H7N7), and their subsequent transmission to humans, have intensified concern over the emergence of a novel strain of influenza with pandemic potential. Three influenza pandemics occurred during the 20th century, with varying degrees of severity; outcomes ranged from the high levels of illness and death observed during the 1918 Spanish flu pandemic (estimates of deaths range from 20 to 100 million [1]) to the much lower levels observed during the pandemics of 1957 and 1968 (≈1 million deaths each [2]). While recognizing that the characteristics of future influenza pandemics are difficult to predict, the World Health Organization (WHO) has recommended that nations prepare pandemic contingency plans (*3*). Several have been drafted, and some have been published (*4**–**7*), although all are subject to continuous refinement. Surveillance, on both a local and global scale, will enable policy makers and practitioners to act during the early phases of a pandemic. However, the likely rapid global spread of a pandemic strain will limit the time available to implement appropriate mitigating strategies, and preemptive contingency planning is needed.

A number of intervention strategies can reduce the impact of influenza pandemics. During interpandemic years, influenza vaccination is used to reduce deaths and disease. However, vaccine is unlikely to be available in time or in sufficient quantities for use during a pandemic (*8**,**9*). Other, nontherapeutic, disease control options may be used, such as those used during the outbreak of severe acute respiratory syndrome (*10*).

However, 2 groups of antiviral drugs are available for the treatment and prophylaxis of influenza. These are the adamantanes (amantadine and rimantadine) and the neuraminidase inhibitors (oseltamivir and zanamivir). The adamantanes may be effective against pandemic strains, but concern exists about adverse reactions and the development of antiviral resistance. Resistance to amantadine has been demonstrated in a number of avian H5 strains (*11*) and its use for treatment of influenza is not recommended (*12*).

The neuraminidase inhibitors (NIs) reduce the period of symptomatic illness from both influenza A and B viruses (*13*) and both are recommended for use in the United Kingdom for treatment of at-risk adults who are able to begin treatment within 48 hours of onset of symptoms. Oseltamivir is also recommended for the treatment of at-risk children >12 months of age (*12*). The development of antiviral resistance has been reported for NIs, particularly related to oseltamivir use in children (*14*), although current evidence suggests that resistant strains are pathogenically weakened (*15*). The use of NIs for treatment of pandemic influenza remains an option since they may improve individual disease outcomes and the effect of the disease in the population.

An influenza pandemic is likely to increase demands on healthcare providers, especially in hospitals. Except in Japan, current levels of NI use are low. Any strategy involving NI use would require stockpiles of these drugs. The potential use of antiviral agents for prophylaxis has been investigated elsewhere and may be of greatest use in the earliest phases of a pandemic to retard the spread of the virus (*16**,**17*). Earlier pandemic influenza modeling studies have also focused on the economic effect of vaccination (*18*) and the use of NI prophylaxis for disease control (*19*). We assessed the potential effect of using NIs for treatment on the estimated number of influenza-related hospitalizations likely to occur during a pandemic. Unlike in previous studies (*20*), we have also taken into account the reduction in infectivity that antiviral treatment may have on community transmission.

## Methods

Our models focused on using NIs to treat different age and risk groups and the potential effects treatment might have on influenza hospitalizations. These effects have been quantified by using the mathematical model described in the Appendix. The length of the latent, noninfectious period was assumed to be 2 days (*19*), and the infectious period was assumed to be 4 days (*19**,**21*). Hospitalization rates for the basline scenario were calculated by using data from interpandemic influenza and are given for different and age risk groups (Table 1).

**Table 1:** Hospitalization rates for clinical patients for different age and risk groups based on data from interpandemic years

Age, y	Hospitalization rates per 100,000	
	High risk	Low risk
<4	3,562	509
5–14	274	39
15–64	873	125
65–74	4,235	605
>75	8,797	1,257

To be effective, NI treatment must be administered within 48 hours of symptom onset. The efficacy of NI treatment appears to prevent 50% of hospitalizations, mirroring efficacy rates against developing complications; this efficacy rate is approximately the same for oseltamivir and zanamivir (*13*). Symptoms were also reduced by ≈1.5 days; treatment was assumed to produce the same decrease in the infectious period.

The population was stratified as for seasonal influenza; persons were considered to be either at high risk for severe outcome or at low risk (*22*). The at-risk group included those with chronic respiratory disease, chronic heart disease, chronic renal failure, diabetes mellitus, and immunosuppression; this group also included all persons living in long-term care facilities, such as nursing homes (*23*), and all those >65 years of age (*24*).

Demographic data used in the model were based on age-specific distribution of the UK population (Office for National Statistics. http://www.statistics.gov.uk). The model was used to simulate a number of scenarios, on the basis of contingency plans and previous pandemics, to investigate the effect of targeting NIs to different age and risk groups on the expected number of hospitalizations during a pandemic.

## Results

The baseline scenario for this study was that advocated by WHO (*3*) and was also used previously by Meltzer et al. (*18*). This scenario assumes a clinical attack rate, in the absence of interventions, of 25% of the population, which occurs during a single wave. Assuming that half of infections are nonclinical or asymptomatic (i.e., a serologic attack rate across the population of 50%) (*25*), a value for the basic reproduction number, *R_0_*, of 1.39 can be calculated. When these parameters are used in the model in the Appendix, the effect of different-sized antiviral stockpiles on the overall clinical attack rate can be estimated.

**Figure 1:**
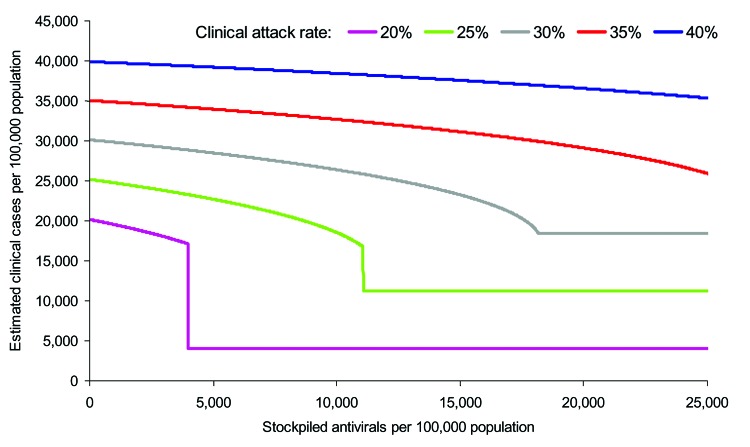
**.** Estimated impact of different sizes of antiviral stockpiles on the number of clinical cases at the end of the pandemic. Depicted are clinical attack rates before interventions of 20%, 25%, 30%, 35%, and 40%, with corresponding values for the basic reproduction number (*R_0_*) of 1.28, 1.39, 1.53, 1.72, and 2.0 respectively. The precipitous decreases observed with the 20% and 25% attack rates result at the points at which the stockpile becomes large enough to last long enough to prevent a recrudescence of the epidemic by suppressing the effective reproduction number.

The outputs from the first set of simulations are shown in Figure 1. The baseline scenario is shown alongside a range of other clinical attack rates (20%–40%) (i.e., varying *R_0_* from 1.28 to 2.0) in the absence of interventions. For these scenarios, antiviral treatment is assumed to be possible within 48 hours of onset for all symptomatic patients until the stockpile is exhausted, with the exception of those <1 year of age, who are not treated at any stage (treatment for this age group is contraindicated [12]). The points on the curves in Figure 1, where the gradients change from vertical to horizontal, indicate the points at which the stockpile is sufficient to treat all patients; increasing the stockpile size would produce no additional benefit and would therefore result in a surplus of antiviral treatments.

For the baseline scenario, a stockpile large enough to treat 12% of the population (i.e., a 12% stockpile) would be sufficient to treat all patients, even if the clinical attack rate in the absence of treatment is 25%. This difference is due to a reduction in the effective reproduction number of the disease, *R_ε_*, caused by shortening the infectious period of those treated by 1.5 days. Across the different attack rates, stockpiles sufficient to treat <1% of the population are unlikely to result in major changes to disease dynamics. Outputs are most sensitive to the clinical attack rate when the reduction in the infection period caused by treatment is sufficient to bring *R_ε_* <1. When *R_ε_* is <1, the number of secondary cases produced by each person is <1, and incidence, therefore, decreases. The value of *R_ε_* can be calculated as



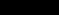



where *S* is the proportion of the population susceptible. With treatment, this equation can be rewritten as



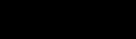



where *I_t_* is the decrease in the infectious period due to treatment, *I_p_* the infectious period, and *c_i_* the proportion of infections in each of the different population subgroups, *i*, that are treated. For the scenarios in Figure 1, *I_t_* = 1.5 days, *I_p_* = 4.0 days and *c_i_* = 0.5 for all groups except those <1 year of age, who only constitute 1.1% of the population. Therefore, the term within the brackets for this scenario can be calculated as 0.81. At the start of the pandemic, *S* is assumed to be 1; therefore, if *R_0_* is <1.23, the outbreak can be controlled by treating all patients. For pandemics in which *R_0_* is >1.23, depletion of susceptible persons through infection is also required before *R_ε_* decreases to <1, which is equivalent to *S* = (0.81*R_0_*) – 1.

**Figure 2:**
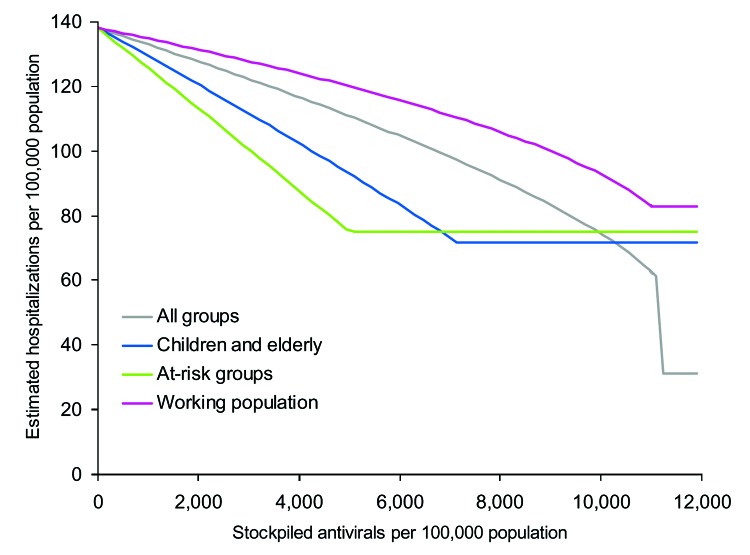
Estimated number of hospitalizations per 100,000 population when different antiviral treatment strategies are applied. Baseline scenario is when the clinical attack rate in the absence of interventions is 25% of the population.

The effect of different treatment strategies on hospitalization rates was generated from the baseline scenario: treating all patients, only at-risk groups, only children and the elderly (1–14 and >65 years of age), and only the working population (15–64 years of age). These scenarios were of potential interest to public health planners; outputs are shown in Figure 2. Given a large enough stockpile, the best option to minimize hospitalizations would be to treat all patients; for this scenario, a 12% antiviral coverage would reduce hospitalizations by up to 77%. An alternative strategy of treating the whole working population reduces the hospitalization rate by up to 40% but requires a similar antiviral stockpile size, and treating the working population consistently fails to reduce the number of hospitalizations below the number that would be expected if everyone were treated, regardless of stockpile size. This increase is because the hospitalization rate for the working population is less than the average in the population and also because treating a smaller proportion of the population has less effect on the overall transmission rate. For stockpile sizes only large enough to treat <5% of the population, the best strategy would be to treat at-risk groups; this strategy is also best for stockpile sizes up to 7%, with hospitalizations at this level reduced by up to 45%. For stockpile sizes from 7% to 10%, the best strategy is to treat children and the elderly (reducing hospitalizations by up to 48%) and for stockpile sizes >10%, to treat everyone.

The optimum treatment strategy is therefore dependent on treating those at highest risk for hospitalization. The simulations for the baseline scenario were based on a uniform age-specific attack rate and on age- and risk-specific hospitalization rates from interpandemic years because of the uncertainty over the precise characteristics of a future pandemic. Since the age-specific clinical attack rate has varied between pandemics, we repeated the analysis above, as far as possible, using the age-specific attack rates from previous pandemics (*26**–**28*) (Table 2) for comparison with the baseline scenario.

**Table 2:** Reported age-specific clinical attack rates (%) for different scenarios

Scenario	Attack rates by age class, y			
	<4	5–14	15–64	>65
Baseline (uniform attack rates)	25	25	25	25
1957 (26)	26	42	22	10
1968 (27)	16	11	49	24
1918 1st wave (28)	16	32	43	9
1918 2nd wave (28)	27	31	29	14
1918 3rd wave (28)	24	22	29	24

**Figure 3:**
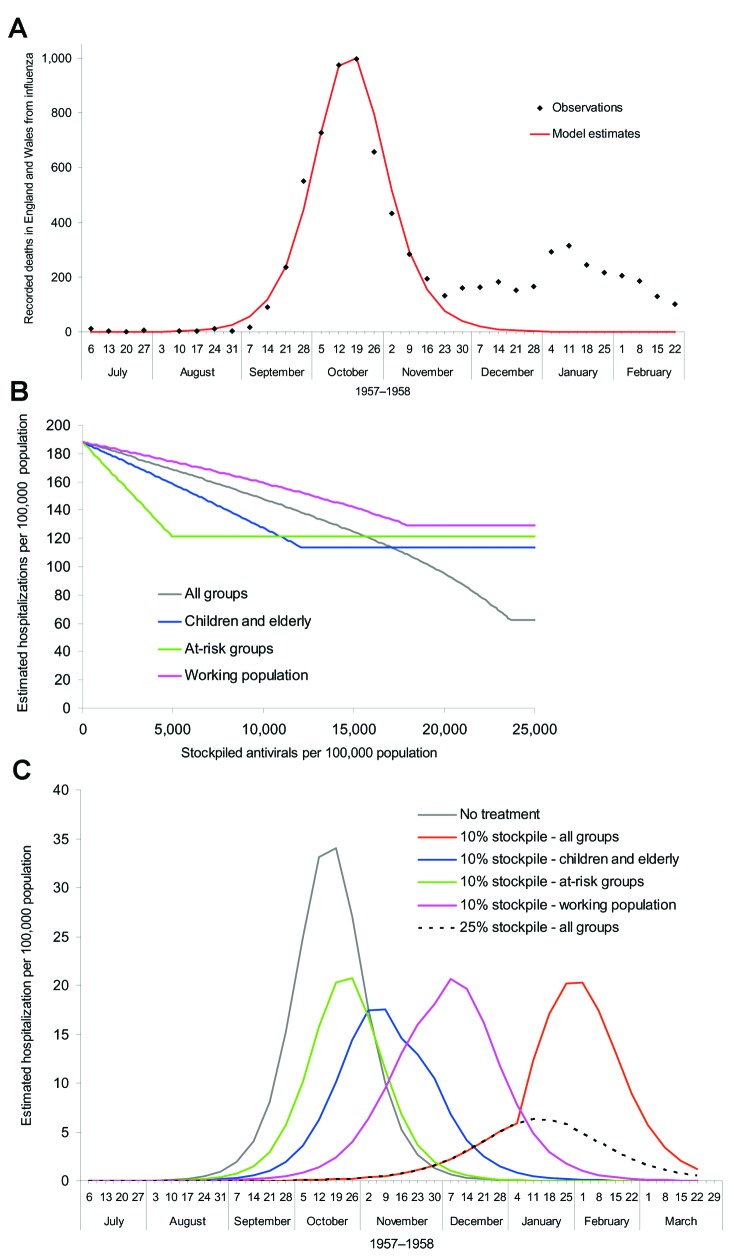
A), Output from the model fitted to the first wave of the 1957 pandemic scaled to fit observations from the 1957 pandemic (*26*). B), Estimated hospitalization rates from a simulated pandemic with available parameters from the 1957 pandemic, as influenced by stockpile size and treatment strategy. C), Impact of treatment strategy on the time course of hospitalizations when the stockpile size is fixed at 10% of the population, the stockpile is fixed at 25% of the population and all clinical cases are treated, and when no treatment is administered.

The 1957 UK pandemic began with imported cases in July 1957; deaths peaked in November 1957, with a reported overall clinical attack rate of 31% (*26*). The proportion of infections resulting in clinical illness was calculated from a small serologic survey of general practitioners; only 46% of the general practitioners surveyed with a positive antibody titer actually had symptoms (*26*). The serologic attack rate was calculated as 67%, which would require *R_0_* = 1.65. The epidemic curve that this figure would generate is shown in Figure 3A, with the curve scaled to fit the 1957 epidemic curve for deaths (*26*). The only additional change from the baseline scenario is the 1957 hospitalization rate, which was reported to be 188/100,000 population (*26*). Using the age-specific attack rates for 1957 (Table 2) in the model, we scaled hospitalization rates to achieve an overall hospitalization rate of 188/100,000 (Table 3).

**Table 3:** Parameters required for scenario specific simulations

	Baseline	1957	1968	1918 1st wave	1918 2nd wave	1918 3rd wave
Overall hospitalization rate per 100,000 population	138*	188†	144†	–	–	–
Overall clinical attack rate, %	25†	31†	21‡	5§	9%§	4§
Overall serologic attack rate, %	50‡	67‡	65§	79§	61§	69§
% immune at start of wave	0¶	0¶	15‡	0¶	0¶	0¶
*R_0_*	1.39‡	1.65‡	2.2‡	2.00†	1.55‡	1.70‡
Case-fatality rate, %	–	–	–	0.70†	3.25†	2.70†

*Derived from model simulations. †Reported values. ‡Calculated directly from data. §Calculated by fitting the model to data. ¶Assumed values.

The results (Figure 3B) show that a 20%–25% antiviral stockpile would be sufficient to treat all patients during the first wave, a figure that is larger than that seen for the baseline scenario, as both the clinical and serologic clinical attack rates were higher. However, qualitatively, the results are similar in spite of the differences in attack rates between different age groups. With a stockpile as large as 20%–25%, an estimated reduction in hospitalizations of ≈67% could be expected. As in the baseline scenario, effective targeting of smaller stockpiles to at-risk groups can also be used to produce large reductions in hospitalization rates. For stockpiles <11%, the best strategy is to treat those at risk, which results in a reduction of 36%. For stockpiles sizes from 11% to 17%, the best strategy is to treat the young and elderly, which results in a 39% reduction. The highest reduction from treating the working population is 31% and remains a suboptimal strategy for any stockpile size.

The implications of different treatment strategies on the hospitalization rates with a 10% stockpile are shown in Figure 3C. Strategies with larger proportions of the 10% stockpile had the greatest effect on the epidemic, steadily delaying, but not diminishing, the peak of hospitalizations. Treating only the working population results in a 15% decrease in hospitalizations, treating all patients results in a 22% decrease, and treating children and the elderly a 32% reduction. With each of these strategies, the antiviral stockpile is exhausted before the end of the pandemic, whereas the fourth strategy of treating at-risk groups reduces hospitalizations by 36% and only requires a 5% stockpile. Therefore, treating those at risk is the most efficient strategy, but further targeting may be considered to avoid surplus treatments.

**Figure 4:**
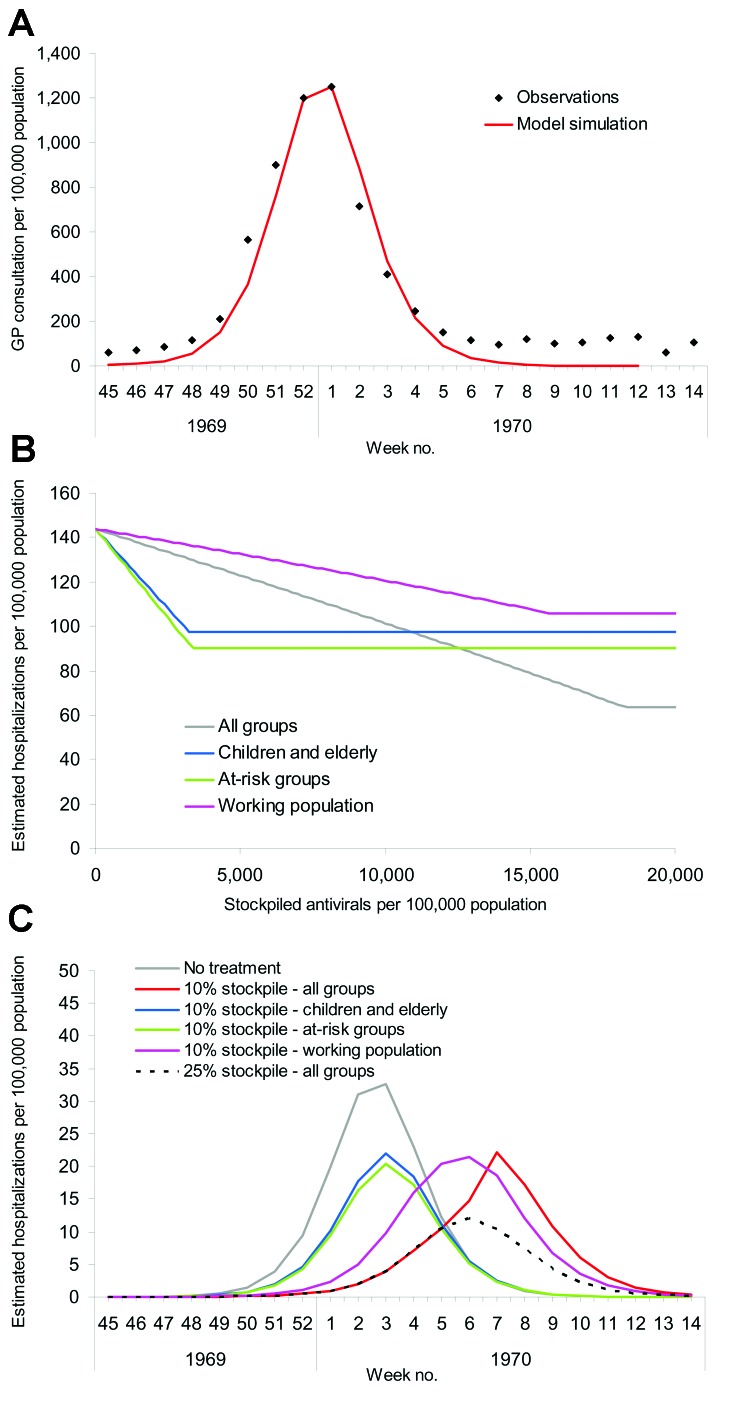
A), Output from the model fitted to the second wave of the 1968 pandemic scaled to fit observations from general practitioners (GPs) from the 1968 pandemic (*29*). B), Estimated hospitalization rates from a simulated pandemic with available parameters from the 1968 pandemic as influenced by stockpile size and treatment strategy. C), Impact of treatment strategy on the time course of hospitalizations when the stockpile size is fixed at 10% of the population, the stockpile is fixed at 25% of the population and all clinical cases are treated, and when no treatment is administered.

The 1968 pandemic was characterized by 2 waves, the first relatively small, occurring from February to April 1969; the larger wave occurred from November 1969 to January 1970 (*27*). We predominately considered the second wave. A confounding factor is that a proportion of the population would have been immune because of the first wave. Weighting age-specific clinical attack rates (Table 1) by age-group sizes from census data, we calculated the overall clinical attack rates for the first and second waves to be 6% and 21%, respectively (27; Office for National Statistics [http://www.statistics.gov.uk]). The serologic attack rate was derived by fitting the model to the data for the second wave from the Royal College of General Practitioners (provided by Douglas Fleming; http://www.rcgp.org.uk); we assumed a similar proportion of asymptomatic cases in both waves. The fit of the model to the data is shown in Figure 4A, from which is derived a 15% residual immunity from the first wave and a 65% serologic attack rate for the second wave, which produces an effective reproduction number of 1.85 for the second wave. The overall hospitalization rate for the second wave was reported as 144 per 100,000 (*29*), and using the age-specific attack rates for 1968 in Table 2, we adjusted the values in Table 1 to fit this value.

The size of the stockpile required to treat all patients is ≈18% (which is relatively small compared to the 1957 pandemic because of the lower clinical attack rate), which leads to fewer patients being treated and less reduction in overall transmission. If all persons whose infections resulted in clinical illness (i.e., patients) were treated, the hospitalization rate would drop by ≈56% (Figure 4B). For the 1968 pandemic, the effects of the different antiviral targeting strategies were different than in the previous scenarios as a result of the different age-specific attack rates, which are shifted more towards the working population (Table 2). Thus, relatively small stockpiles are required to treat either the at-risk group or the young and elderly group (≈3% for each group), since most patients are in the working population and neither of these 2 groups. For stockpiles of up to 12%, treating the at-risk group is marginally better than treating the young and the elderly (37% reduction in hospitalization as opposed to 32%), and for stockpiles >12%, treating all clinical patients would be the best strategy.

The effects of the different treatment strategies with a 10% stockpile are shown in Figure 4C. Hospitalizations would drop by ≈29% if all patients were treated and by 16% if the working population were treated; both treatment strategies would lead to the stockpiles' being exhausted. As above, treating those at risk would reduce hospitalizations by 37%, whereas treating only children and the elderly would reduce hospitalizations by 32% and only require a 3% stockpile per group. Of these 4 strategies, treating the at-risk groups is the most efficient, but given surplus stockpile, further extension of the groups to be targeted may be considered.

**Figure 5:**
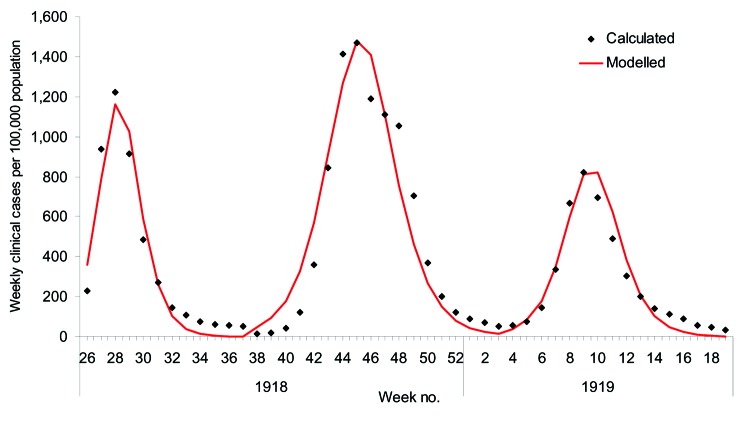
Clinical cases per week estimated by using the clinical case-fatality rates and weekly mortality statistics for the 1918 pandemic and by fitting the basic reproduction number (*R_0_*) to data from each of the waves by using the transmission model (*28*).

The characteristics for the 1918 pandemic differ substantially from the other 2 in that 3 distinct waves occurred; the age-specific attack rates were highest for those in their teens, 20s, and 30s; and the mortality rates were higher (*2*). In addition, age-specific attack rates and mortality rates differed for each of the 3 waves (*28*). Modeling based on the 1918 pandemic was therefore considerably less straightforward than for the previous 2 pandemics, and an approach was taken to fit the transmission model to each of the 3 waves, separately. No cross-immunity was assumed between different waves since studies suggested only weak effects; indeed, some studies suggested greater susceptibility in the third wave if a person had had influenza in the first pandemic wave (*28*). Clinical attack rates were calculated from reported weekly mortality data and clinical case-fatality rates (*28*). Serologic attack rates were then fitted separately to each of the curves (Figure 5), from which values of *R_0_* = 2.0, 1.55, and 1.7 were derived from each of the respective waves. The estimate for the second wave is lower than other estimates of ≈3 (*30*) derived from US cities and is probably because our estimates were derived from data from throughout England and Wales, thereby incorporating spatial heterogeneity.

Since hospitalization rates were not available for any of the 3 waves, we considered the effect of antiviral treatment on death. The potential efficacy of antiviral treatments in preventing death between waves may have differed, but it was assumed to provide 50% protection against death. This estimate was based on the assumption that 50% protection from the more serious outcomes of influenza can be translated to equivalent protection from death (*20*).

**Figure 6:**
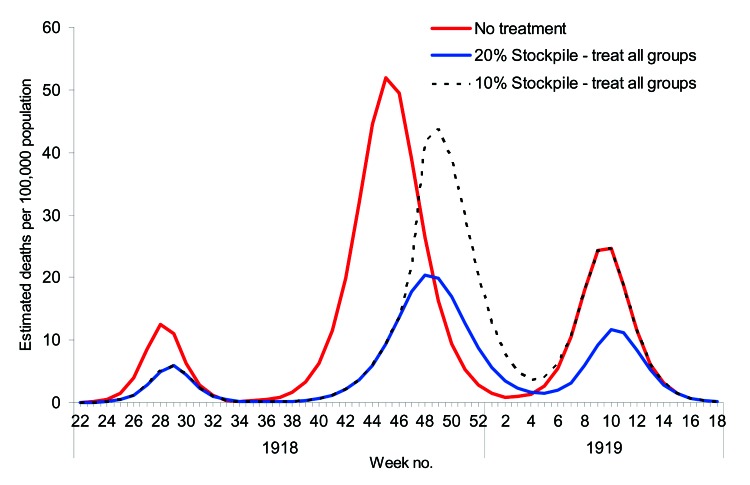
Estimated number of deaths from the 3 waves of the 1918 pandemic when there is no treatment, a 20% antiviral stockpile, and a 10% antiviral stockpile.

A pandemic with the characteristics of that in 1918 would, without antiviral treatment, produce an estimated number of deaths equivalent to ≈0.5% of the population across all 3 waves. However, a 20% stockpile sufficient to treat all patients across the 3 waves would result in ≈53% reduction in deaths. With a smaller stockpile of 10%, the reduction in deaths was only 17% because the stockpile becomes exhausted during the second wave, before most of the deaths occur (Figure 6).

## Discussion

The baseline scenario with an overall clinical attack rate of 25%, as currently advised by WHO (*3*), is roughly in accordance with data from previous pandemics. The general conclusion from our study is that antiviral treatments for 20% to 25% of the population are likely to be sufficient to treat all patients for pandemics with characteristics that have been observed to date. The size of the stockpile required will depend on the clinical attack rate of the pandemic and the *R_0_* value.

However, with smaller stockpile sizes, substantial reductions in hospitalizations can be achieved through targeting. For the smallest stockpiles, the best strategy was to treat conventional influenza at-risk groups. Treating the young and elderly is only slightly less effective. Treating the working population may have benefits beyond reducing hospitalizations, such as reducing illness-related absenteeism, but it consistently fails to be the best strategy for reducing hospitalizations. For large stockpiles, treating all patients is consistently the best strategy in reducing hospitalization and transmission. When all patients are treated, the marginal effect of treatment on reduced transmission increases with the number of patients treated, until all patients have been treated.

Further studies regarding the effects of antiviral treatments would improve the robustness of the parameter estimates. In particular, better estimates on the efficacy of NI treatment against hospitalization and death rates for different age and risk groups and estimates on the reduction in the infectious period are required. Also, the issue of antiviral resistance needs to be resolved since it could compromise NI effectiveness.

The scenarios above assume that clinical patients were treated within 48 hours of onset of symptoms; however, in reality, some cases will be diagnosed or reported too late, and other patients will be administered drugs mistakenly. To maximize the benefits of antiviral treatment, patients should be strongly encouraged to seek treatment and treatment should be supported by sound clinical judgment and diagnostic capability. If high levels of treatment are not achievable, disproportionately higher hospitalization rates than those calculated here would ensue. In addition, identifying groups with higher transmission rates for targeting treatment would result in greater reductions in transmission than reported here.

Assessments will need to be recalculated in the earliest phases of a pandemic with real-time data to confirm or update the assumptions used and ensure that the model parameters are appropriate. Therefore, were a pandemic to occur, intensive analysis of its dynamics would be required at its start.

## Appendix

### Mathematical Model Used To Calculate Outputs

The model used was based on Kermack and McKendrick (*31*), which has formed the basis of a number of models for both epidemic and pandemic influenza (*32**–**34*) and is implemented by using the set of differential equations given in equation 1.


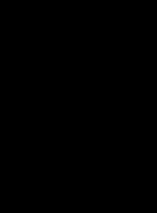
 equation 1

where *α* = 1/L = 0.5, *γ* = 1/PP = 0.4, λ = 1/IP = 2/3, and *β* = *R_0_*/(PP + IP). LP represents the length of the latent period; PP represents the length of the nonsymptomatic infectious period; and IP represents the length of the infectious symptomatic period. *S* represents the total proportion susceptible, *E* the total proportion incubating, *P_i_* the proportion from the total population in each group *i* within the first 2.5 days of their infectious period, *I_i_* the proportion of total population in each group *i* within the final 1.5 days of their infectious period, and *R* the total proportion recovered and immune or dead. *c_i_* is the proportion of infections resulting in clinical cases, and *T_i_* is the proportion of group *i* receiving treatment. The average number of secondary cases per primary case when the population is entirely susceptible is represented by *R_0_*_,_ and the proportion of the population in each group *i* is given by *N_i_*. The proportion treated in each group each week can be given as *A_i_*(*t*) as



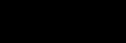



where *t* is time in days.

The proportion of the population within each group being hospitalized each week, *Hi*(*t*), can be calculated as



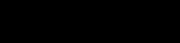



where ε is the efficacy of antiviral treatment against hospitalization and *h_i_* is the hospitalization rate for each group *i*.

Supplementary information on the probability of hospitalization in the absence of vaccination is available from the author (see Comments to the Authors).
